# A rare case report: Spontaneous rectus sheath and iliopsoas hematomas: Clinical presentation, management, and implications

**DOI:** 10.1016/j.ijscr.2023.108756

**Published:** 2023-08-29

**Authors:** Sana Landolsi, Mohamed Raouf Ben Othmane, Med Dheker Touati, Ahmed Omry, Fahd Khefacha, Faouzi Chebbi

**Affiliations:** a General Surgery Department, Mahmoud El Matri Hospital, V59M+628, Ariana, Tunisia; b Faculty of Medicine of Tunis, University of Tunis El Manar, R534+F9H, Rue de la Faculté de Médecine, Tunis, Tunisia

**Keywords:** Case report, Rectus sheath hematomas, Surgery, Emergency, Spontaneous hematoma, Mesh

## Abstract

**Introduction and importance:**

Frequently misdiagnosed, the clinical condition of soft tissue hematoma typically emerges following blunt abdominal trauma and/or anticoagulant medication usage, with spontaneous occurrences being infrequent. In this case report, we present a spontaneous rectus sheath and iliopsoas hematomas without obvious classical risk factors. The purpose of presenting this case is to bring attention to this unusual clinical condition and emphasize the role of thorough history and physical examination in determining the suitable course of treatment.

**Case report:**

A 50-year-old woman with no medical history presented at the emergency room due to sudden asthenia and abdominal pain. Physical examination revealed a painful 20 cm hypogastric mass and left lumbar swelling causing leg bending. Lab tests indicated anemia and normal coagulation. CT scans showed significant hematomas in the left rectus and psoas muscles. Intensive monitoring and supportive measures stabilized her condition without resorting to surgery.

**Clinical discussion:**

Soft tissue hematomas, notably in the rectus sheath or iliopsoas muscle, are rare but potentially severe conditions. Their pathophysiology is not fully understood, and risk factors include age, anticoagulant use, and comorbidities. Diagnosis involves abdominal examination, anemia, and CT findings. Management varies based on symptom severity and blood loss, ranging from conservative approaches to surgery or embolization.

**Conclusion:**

Swift identification and effective handling of soft tissue hematomas hold utmost importance. The thorough history-taking and comprehensive physical examination play pivotal roles within this protocol. While supportive care constitutes the primary mode of management, instances arise where surgical intervention or vascular embolization becomes imperative for hematomas unresponsive to treatment and presenting hemodynamic instability.

## Highlights

• **Uncommon Spontaneous Hematomas**: An uncommon occurrence of spontaneous rectus sheath and iliopsoas hematomas.
• **Diagnostic Challenges and Multidisciplinary Approach:** The article highlights the diagnostic challenges posed by soft tissue hematomas.
• **Successful Conservative Management:** The study showcases a successful conservative management strategy for spontaneous hematomas.

## 1 Introduction

Hematoma in the iliopsoas or rectus sheath is an infrequent issue that holds the potential for life-threatening consequences [1,2]. In the absence of an apparent cause, its diagnosis is challenging [3]. Herein, we present a case of spontaneous rectus sheath and iliopsoas hematoma without evident risk factors. Furthermore, we delve into the existing literature concerning diagnostic approaches and treatment strategies.

This work has been reported in line with the SCARE 2020 criteria [4].

## 2 Case presentation

A 55-year-old female, a homemaker with no prior medical or surgical history, and no history of antiplatelet or anticoagulant use, presented at the Mahmoud El Matri Hospital in Ariana, Tunisia, at the emergency care clinic with asthenia and abdominal pain of one-day duration. She reported the sudden onset of a hypogastric swelling that had been evolving for the past two days. The thorough history did not uncover fever, nausea, vomiting, changes in bowel habits, cough, trauma, or minor concussion. Symptoms appeared suddenly and acutely after lifting a heavy object.

**Fig. 1:**
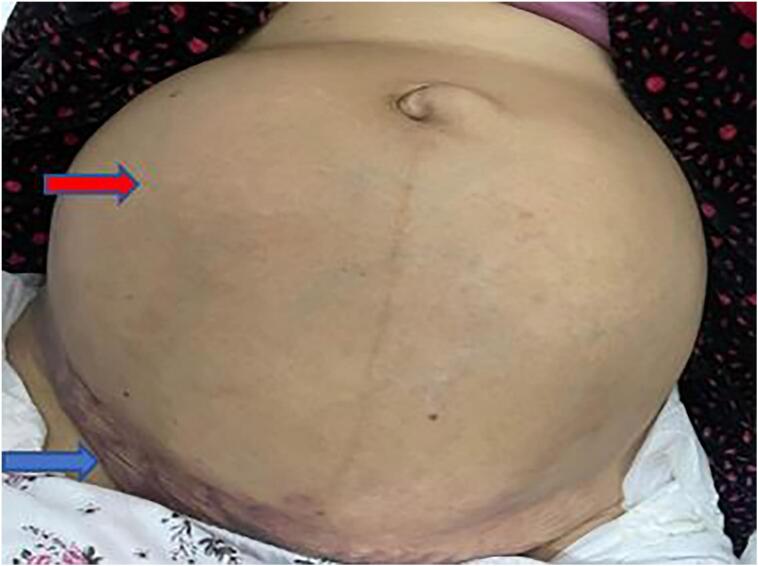
Abdominal mass (red arrow), subcutaneous extension of the hematoma (blue arrow). (For interpretation of the references to colour in this figure legend, the reader is referred to the web version of this article.)

Upon physical examination, an elevated heart rate of 105 beats per minute and stable blood pressure of 120/75 mmHg were observed. The respiratory rate was normal. Cutaneous pallor of the mucous membranes was also noted. During the abdominal examination, a hypogastric mass measuring 20 cm in its largest dimension was palpated (Fig. 1), and it was sensitive to touch. Additionally, another left lumbar mass of 10 cm was present, causing an irreducible leg bending.

Laboratory data revealed acute anemia with a hemoglobin level of 5 g/dL and hematocrit levels at 17.5 %. Blood group was A positive. Hemostasis assessment fell within the normal range. Serum electrolyte and kidney function test results showed no abnormalities.

Regarding the patient's hemoglobin level, it's important to consider whether bleeding occurred at other sites that might have contributed to the hemoglobin level reaching 5 g/dL. We thoroughly explored potential sources of bleeding, including transrectal, vaginal, and upper gastrointestinal bleeding, and found no abnormal findings.

**Fig. 2:**
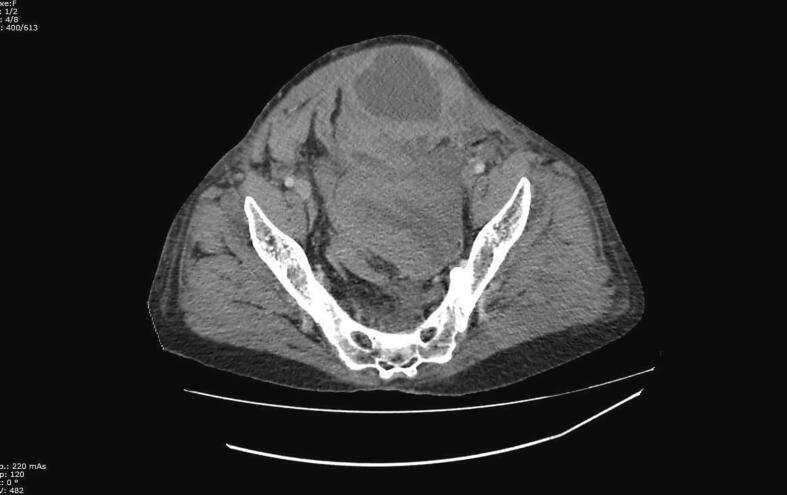
Axial section of a CT scan showing the large hematoma in the left rectus muscle.

**Fig. 3:**
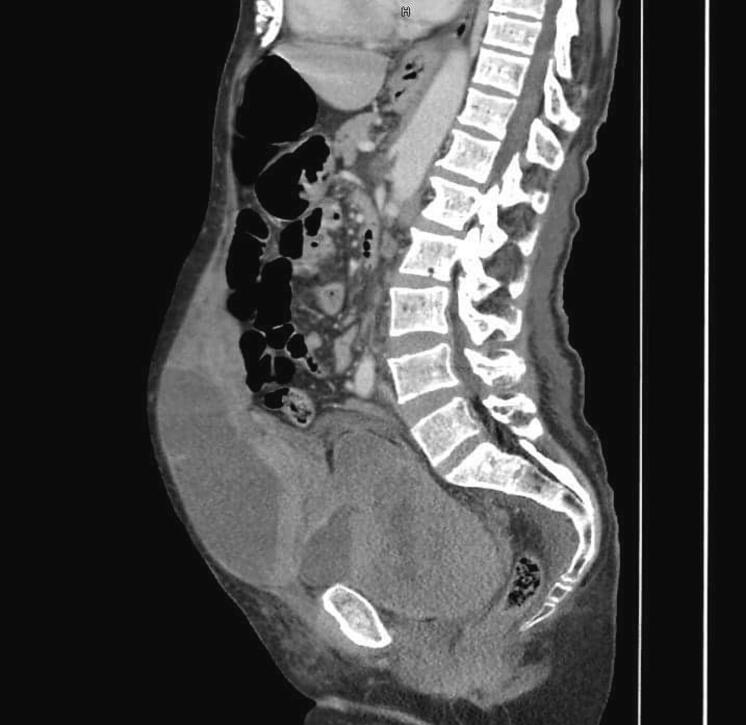
Sagittal section of a CT scan showing the second hematoma in the left psoas muscle.

An emergency computed tomography (CT) scan was performed, revealing a large hematoma in the left rectus muscle that communicated with the preperitoneal space and the ipsilateral para-rectal fossa. The hematoma measured 185 mm by 93 mm in axial dimensions and 220 mm in sagittal dimensions. Its density was heterogeneous with areas of spontaneous hyperdensity not enhanced and hypodense zones. The pelvic organs were displaced to the right side (Fig. 2). Additionally, a second hematoma was identified in the left psoas muscle, with the same appearance as the first one, measuring 50 mm by 37 mm by 125 mm. There was no extravasation of contrast media (Fig. 3). It's important to note that, due to the unavailability of angiography at our hospital, further assessment through this method was not conducted.

The patient received transfusions of packed red blood cells and fresh frozen plasma with a positive transfusion response. The course was marked by stable hemodynamic status, and the post-transfusion hemoglobin level reached 10 g/dL (after 10 units of packed red blood cells and 5 units of plasma). It was decided to proceed with observation.

The patient was discharged following a gradual recovery of leg function with the assistance of physical therapy. In the one-month follow-up, there were no unfavorable outcomes. Additionally, upon discharge, the patient was referred for further hematological evaluation to eliminate the possibility of an underlying cause for this hematoma, particularly a rare coagulopathy. The hematological assessment, however, did not identify any coagulopathy or other factors that could explain the occurrence of this hematoma.

## 3 Discussion

We presented a successful case of conservative management for spontaneous iliopsoas and rectus sheath hematomas resulting in acute anemia. The primary highlight of our study lies in the prompt diagnosis and effective conservative approach, facilitating the patient's recovery without resorting to surgery. However, a notable limitation is the relatively short duration of follow-up.

Soft tissue hematomas can manifest in various locations, although they are most commonly observed within the rectus sheath or iliopsoas muscle [5].

Spontaneous iliopsoas hematoma is an uncommon condition, with an estimated prevalence ranging from 0.6 % to 6.6 % [2]. The incidence of spontaneous soft tissue hematoma is unknown due to its rarity. The number of new cases diagnosed worldwide each year, over five years, or per decade is also unknown, but it is likely to be small.

Rectus sheath hematoma (RSH) is typically seen in the context of blunt abdominal trauma and/or anticoagulation therapy, rarely occurring spontaneously [1,3,6]. This condition lacks comprehensive research, and its underlying physiological mechanisms remain unclear [2].

Coagulopathy caused by hemophilia or the increasing utilization of anticoagulant and antiplatelet treatments could potentially contribute to the higher incidence of reported cases of soft tissue hematomas [5]. The prevalent risk factors that have been identified include age, the use of anticoagulants, elevated body mass index (BMI), and concurrent medical conditions like hypertension and diabetes [5]. Intense and excessive contraction of the rectus abdominis or direct trauma to the abdominal area can result in bleeding and hemorrhage due to the rupture of one of the epigastric arteries and/or the rectus abdominis muscle [6]. Moreover, there is a hypothesis suggesting heightened vulnerability to the rupture of pre-existing retroperitoneal microvascular atherosclerosis during minor occurrences like coughing, vomiting, or even changes in posture, which can result in retroperitoneal bleeding [5].

Clinical symptoms are nonspecific [2,3]. The primary manifestation is an acute onset of abdominal pain. Associated clinical features depend on the onset and size of the hematoma. Patients may present signs of neurological compression in the same-sided lower limbs or just general manifestations of hypovolemia [3]. Conversely, anemia is consistently observed such as our patient [3]. Opting for a CT scan of the abdomen is preferred as it provides crucial information regarding the hematoma, including its precise dimensions, extension, and any indications of contrast leakage that could suggest ongoing bleeding [2,3]. Although, MRI and ultrasound can be modalities of choice [2].

The treatment approach depends on the extent of hemorrhaging, the patient's hemodynamic status, and the presence or absence of neurological deficits [3,6]. There is no consensus regarding the preferred approach, surgery or embolization, to adopt towards an active bleeding [2,6].

Surgical intervention is linked to notable morbidity given the advanced age and presence of multiple comorbidities in these individuals [3]. Coil or gel foam embolization of the epigastric arteries has proven effective in managing patients who experience persistent bleeding even after coagulopathy has been reversed [[1], [2], [3]].

For patients with mild symptoms, conservative management involving bed rest, blood transfusion, and potentially administering anticoagulant antagonist medications is usually adequate [2,3]. Spontaneous complete resolution, especially in large hematomas, may take up to several months [6].

## 4 Conclusion

We present this case due to its uncommon occurrence among spontaneous hematomas. Its uniqueness lies in the absence of typical factors like anticoagulants and trauma. The presentation with atypical acute anemia underscores the importance of early diagnosis, and the effective conservative management highlights minimally invasive approaches.

Spontaneous soft tissue hematomas are a potentially life-threatening condition [2]. Given their rarity and lack of distinctive signs, especially in patients without a medical history, their diagnosis is challenging [2,3]. Timely recognition is crucial for guiding suitable therapeutic strategies, as showcased in our case where prompt diagnosis and conservative intervention led to successful recovery without resorting to surgery.

## Patient consent

Written informed consent was obtained from the patient for the publication of this case report and its accompanying images. A copy of the written consent is available for the Editor-in-Chief of this journal to review upon request.

## Grant information

The author(s) declared that no grants were involved in supporting this work.

## Declaration of generative AI in scientific writing

AI tools were not used for the elaboration of the manuscript.

## Ethical approval

Ethical approval is not applicable/waived at our institution.

## Funding

This research did not receive funding from any specific grant provided by public, commercial, or not-for-profit organizations.

## Author contribution

**Med dheker touati** and **Sana landolsi** contributed to manuscript writing andediting, and data collection; **Med raouf ben othmane** and **ahmed omry** contributed to data analysis;

**Fahd Khefacha** and **Faouzi chebbi** contributed to conceptualization and supervision;

All authors have read and approved the final manuscript.

## Guarantor

Dr. Med Dheker Touati.

## Research registration number

1. Name of the registry: N/A.
2. Unique identifying number or registration ID: N/A.
3. Hyperlink to your specific registration (must be publicly accessible and will be checked): N/A.

## Conflict of interest statement

No conflicts of interest.
